# Direct Filtration of Mine Water with High-Concentration Fine Suspended Solids Using Flat-Sheet PolyCera Membranes: Performance, Fouling, and Cleaning

**DOI:** 10.3390/membranes16100336

**Published:** 2026-10-10

**Authors:** Xinhao Xu, Bo Chang, Zhipeng Su, Zhihui Wang, Xuwen He

**Affiliations:** 1 School of Chemical and Environmental Engineering, China University of Mining and Technology (Beijing), Beijing 100083, China; 15965538261@163.com (X.X.); 11432508@chnenergy.com.cn (B.C.); su.567@outlook.com (Z.S.); wzhzoo@sina.com (Z.W.); 2 CHN Energy Xinjiang Energy and Chemical Co., Ltd., Urumqi 831400, China; 3 Xinjiang Xintou Coal Industry Co., Ltd., Urumqi 830017, China

**Keywords:** mine water with high-concentration fine suspended solids, flat-sheet PolyCera membrane, direct filtration, membrane fouling mechanism, composite chemical cleaning agent

## Abstract

As coal mining intensity increases, the concentration of fine suspended solids (<10 μm) in mine water continues to rise. These particles carry a highly negative surface charge and interact synergistically with emulsified oil, making them difficult to settle spontaneously under natural conditions. For the treatment of such water, the conventional three-stage process of “coagulation–sedimentation–sand filtration–ultrafiltration” suffers from high chemical consumption and a long treatment train. In this study, a submerged flat-sheet PolyCera membrane direct filtration process was developed. Through parameter optimization, membrane fouling mechanism analysis, and cleaning strategy development, its treatment performance was systematically evaluated. The results showed that the produced water turbidity remained stably below 1 NTU, demonstrating effective retention of fine suspended solids. Membrane fouling was dominated by cake-layer blocking, in which inorganic minerals formed a rigid skeleton and emulsified oil is proposed to act as an organic binding medium. The combined cleaning strategy of “aeration + pure water backwashing” and a composite cleaning agent achieved a membrane flux recovery rate of more than 95%. This process shows the potential to simplify the conventional three-stage treatment flow under the tested conditions, although site-specific water quality and operational conditions should be considered, providing technical support for the treatment of similar mine water.

## 1. Introduction

Coal is the dominant energy source in China and plays a vital role in safeguarding national energy security. As coal mining advances rapidly toward mechanization, automation, and refined operation, mining intensity has increased markedly, and mine water discharge has continued to rise [1,2], making the demand for resource utilization increasingly urgent. However, coal fines and rock dust generated by mechanical crushing, roof caving, and other mining-induced processes enter mine water in large quantities. In addition, fly ash and crushed coal gangue in underground backfill mining release elements such as Si, Al, and S during long-term immersion and leaching. These inputs, together with emulsified oil contamination from hydraulic equipment leakage, give suspended solids in mine water combined contamination characteristics of high concentration, fine particle size, high negative charge, and oil content [3,4,5]. The average particle size of these suspended solids is only 5–10 μm, and their surface zeta potential is as low as −30 to −40 mV. They are difficult to settle spontaneously under natural sedimentation conditions, which severely limits the efficiency of mine water resource utilization.

For the treatment of mine water with high suspended solids, existing processes can be mainly classified into three categories according to their separation driving forces and mechanisms: chemical coagulation, physical enhancement, and membrane separation.

Chemical coagulation (e.g., “coagulation–sedimentation–sand filtration”) is currently the most widely used process route. Its core mechanism involves compression of the electrical double layer by coagulants and bridging flocculation by flocculants, which destabilize and aggregate fine particles into larger flocs, followed by gravity settling for solid–liquid separation [6,7,8]. However, this type of process faces significant bottlenecks when treating mine water with high concentrations of fine suspended solids. Literature reports indicate that when the zeta potential of raw water is in a highly negative range, large amounts of coagulants are required to neutralize the surface charge of particles. The polyaluminium chloride (PAC) dosage can reach 250 mg/L, approximately 3–4 times the level used in conventional mine water treatment. This leads to significantly increased chemical costs and substantial chemical sludge production. Meanwhile, the flocs formed by fine particles are small in size and low in density, and their gravity settling velocity is extremely slow, requiring a long hydraulic retention time [9]. Inclined plate settlers frequently become clogged owing to the adhesion of oil–solid composite pollutants, and quartz sand filter beds harden because of the adhesive effect of oils. Conventional hydraulic cleaning cannot effectively regenerate them, and the filtration cycle is substantially shortened.

To overcome the rate limitation of gravity sedimentation, physically enhanced processes (e.g., high-gradient magnetic separation and heavy-medium rapid sedimentation) introduce magnetic seed particles or heavy media and, on the basis of coagulation–flocculation, couple an external magnetic field or an enhanced gravity field, thereby increasing the floc settling velocity by tens to hundreds of times [10,11,12,13,14,15,16]. The hydraulic retention time can be greatly shortened to the order of several minutes, showing the advantage of high treatment efficiency for conventional mine water with high suspended solids. However, such processes still inherently depend on the addition of coagulants and flocculants to form flocs that are sufficiently large and dense to be effectively captured by physical fields; thus, the problem of chemical consumption is not eliminated. In addition, their separation efficiency is significantly affected by floc size. For fine suspended solids with an average particle size < 10 μm and emulsified oil pollutants, the capture capacity of physical fields decreases substantially, and the effluent quality cannot stably meet reuse standards. Therefore, although physical enhancement shortens the settling time, it does not fundamentally overcome the limitations of chemical dependence and insufficient removal efficiency for fine particles.

Membrane separation provides a new pathway for physical screening without the addition of coagulants or flocculants. Tubular ceramic membranes, owing to their advantages such as fouling resistance and long service life, have been explored for application in some mining areas. This process achieves solid–liquid separation through membrane pore interception without the addition of coagulation chemicals, thereby avoiding chemical sludge production and chemical costs at the source [17,18,19,20]. However, in practical operation, it has been found that the synergistic effect of three types of pollutants—high-concentration fine coal fines, rock dust, and fibers—causes membrane flux to decline sharply within several hours. Physical backwashing cannot effectively restore the flux, and frequent chemical cleaning is required, which severely restricts long-term operational stability [21,22]. In recent years, PolyCera, as a new type of polymeric ceramic membrane material, has shown application potential in the field of mine water treatment by combining the fouling resistance of ceramic membranes with the flexibility of organic membranes. Compared with tubular ceramic membranes, PolyCera membranes have smoother surfaces and better fouling resistance. Previous studies have preliminarily confirmed the feasibility of direct filtration of conventional mine water using PolyCera membranes. For example, Wang et al. [23] used spiral-wound PolyCera membranes for direct filtration of conventional mine water, focusing on membrane fouling and cleaning. However, systematic research is still lacking on membrane fouling behavior and cleaning strategies under combined pollution by high-concentration fine suspended solids and emulsified oil.

In summary, existing chemical coagulation processes suffer from slow settling rates and high chemical consumption. Although physically enhanced processes accelerate settling, their dependence on chemicals remains unresolved. Tubular ceramic membranes require no chemical addition, but they exhibit rapid flux decline and require frequent cleaning. Therefore, there is an urgent need to develop a novel membrane separation process with a short flow, no chemical addition, and excellent fouling resistance. This study aims to develop a submerged flat-sheet PolyCera membrane direct filtration process, systematically optimize key operating parameters, reveal its membrane fouling mechanisms, and establish a targeted composite cleaning strategy. The goal is to develop a short-process direct filtration alternative that has the potential to replace or simplify the existing three-stage treatment process of “coagulation–sedimentation–sand filtration–ultrafiltration,” thereby providing technical support for the resource utilization of mine water with high-concentration fine suspended solids.

## 2. Materials and Methods

### 2.1. Characteristics of the Raw Mine Water

The concentration and particle size of suspended solids in mine water are affected by factors such as mining activity intensity and water inflow conditions during different periods [24], resulting in a wide fluctuation range and drastic changes in water quality. The raw water was obtained from coal mine water characterized by high concentration and fine suspended solids, and the main water quality parameters are presented in Table 1.

Figure 1 presents the cumulative particle size distribution curves of suspended solids during three periods with different mining intensities (Period 1: peak mining period; Period 2: shutdown period; Period 3: normal mining period). Overall, the suspended solids in all three periods were dominated by fine particles (<10 μm), with a relatively low proportion of coarse particles. In terms of period comparison, the proportion of fine particles was highest during the peak mining period, reflecting further particle refinement caused by mechanical crushing under high-intensity disturbance. In contrast, the curve for the shutdown period shifted to the right, indicating that after coarse particles gradually settled, the proportion of fine particles in the residual suspended solids increased compared with the normal mining period. However, regardless of fluctuations in mining intensity, all three periods were dominated by fine particles. Fine particles have an extremely low natural settling rate [25], which is one of the important reasons for their poor settleability.

As shown in Figure 2, compared with conventional mine water, the pH–zeta potential curve of the difficult-to-settle mine water exhibited notable specificity: under natural pH conditions (6.0–9.0), the zeta potential was lower, and the isoelectric point (IEP) shifted markedly toward strongly acidic conditions, far away from the natural pH range. This is attributed to the oil–solid composites formed by coal fines, rock dust, and emulsified oil in the suspended solids. The oil substances adsorbed on their surfaces form an organic film layer on the particle surfaces, enhancing steric hindrance repulsion. Together with the high negative charge, this constitutes an electrostatic–steric dual mechanism that keeps the particles highly dispersed and makes spontaneous aggregation and settling difficult under natural conditions [26,27]. This constitutes the chemical essence of the “difficult-to-settle” characteristic of this type of mine water.

As shown in Table 2, coal fines accounted for the highest proportion (44.3%) in the mine water with high-concentration fine suspended solids. Their hydrophobic surfaces readily adsorb emulsified oil to form oil–solid composite particles, thereby enhancing interparticle adhesion. Rock dust ranked second in proportion (36.1%). However, the XRD pattern of the raw water in Figure 3 shows that it is mainly composed of minerals such as quartz and kaolinite. Its surfaces carry abundant negative charges and are the main source of the highly negative zeta potential of the suspension system. Although the proportion of fibers was relatively low (15.7%), fibers readily adsorb micro-bubbles, emulsified oil, and fine coal fines and rock dust in water, forming composite floc structures that are difficult to settle naturally because of their filamentous morphology.

Based on the above analysis, the suspended solids in this mine water are concentrated in the micron-sized fine particle range (<10 μm). Their zeta potential is low, and the isoelectric point is far from the natural pH. The combination of these factors makes both gravity settling and spontaneous aggregation difficult to proceed effectively. Although rock dust accounts for a lower proportion than coal fines, it is the main source of the high negative charge, and the electrostatic repulsion field it induces keeps the suspended solids in a highly dispersed and stable state. Under the combined action of these three components, the mine water exhibits difficult-to-settle characteristics.

### 2.2. Membrane Material

The membrane used in the experiment was a flat-sheet PolyCera membrane with a pore size of 50 nm, a molecular weight cut-off (MWCO) of 250 kDa, an effective membrane area of 0.36 m^2^, and an operating pH range of 1–12. It exhibits high mechanical strength, superhydrophilicity, and excellent oil and fouling resistance.

The membrane cleaning agents used were an acidic cleaning agent (HCl solution, 36%, Sinopharm Chemical Reagent Co., Ltd., Shanghai, China.), an alkaline cleaning agent (NaOH solution, ≥96%, Sinopharm Chemical Reagent Co., Ltd., China.), an oil-fouling cleaning agent. The composite cleaning agent consisted of 0.5 wt% NaOH, 0.7 wt% 1-hydroxyethylene-1,1-diphosphonic acid (HEDP, 50%, Shandong Yukang Chemical Co., Ltd., Jinan, China.), and 0.5 wt% alkyl amidopropyl hydroxyisutaine (HSB, 35%, Guangzhou Zhonghai Chemical Co., Ltd., Guangzhou, China.).

### 2.3. Membrane Filtration Systems

The experimental setup was a custom-designed membrane filtration system, and its schematic diagram is shown in Figure 4.

The system consisted of the following components: a feed pump, a raw water tank, a produced water storage tank, a flat-sheet membrane module, a produced water pump, a sludge discharge pump, a cleaning water tank, and various valves. During operation, valves V-1 and V-2, the feed pump, and the produced water pump were kept open. Raw water was used as the water source. Raw water was delivered by the feed pump to the feed tank, and the permeate side was maintained at atmospheric pressure. The TMP was therefore approximately equal to the feed pressure, which was measured by a pressure gauge installed on the feed side. The filtered produced water was transported to the produced water storage tank, and part of the concentrate was discharged, as shown in Figure 4a. After a period of operation, pollutants accumulated on the membrane surface, and cleaning was required. During backwashing, valve V-4 and the produced water pump were opened. At this time, water in the cleaning water tank was pumped into the membrane by the produced water pump for soaking and cleaning, as shown in Figure 4b. The lab-scale filtration experiments were performed as single runs because the available mine water sample volume was limited and insufficient for independent replicates.

In the long-term validation system, the membrane module was submerged in an open feed tank. The feed side was at atmospheric pressure, and the permeate side was drawn by a suction pump. The transmembrane pressure was directly measured by a vacuum/pressure sensor installed on the permeate side.

### 2.4. Calculation of Membrane Parameters

The membrane flux, *J_t_*, is defined by Equation (1):

(1) $$J_{t}=\frac{V}{At}$$

where *J_t_* is the membrane flux at time *t* (L·m^−2^·h^−1^); *V* is the volume of fluid passing through the membrane (L); *A* is the effective filtration area of the membrane (m^2^); and *t* is the filtration time (h).

The specific flux, *J_R_*, is defined by Equation (2):

(2) $$J_{R}=\frac{J_{t}}{J_{0}}$$

where *J_R_* is the specific flux at time *t* (%); *J_t_* is the membrane flux at time *t* (LMH); and *J*_0_ is the initial membrane flux (LMH).

The flux recovery rate, *F_RR_*, is defined by Equation (3):

(3) $$F_{RR}=\frac{J_{w}}{J_{0}}\times 100\%$$

where *F_RR_* is the flux recovery rate (%); *J_w_* is the membrane flux after cleaning (LMH); and *J*_0_ is the initial membrane flux (LMH).

The total membrane resistance, *Rt*, is defined by Equation (4):

(4) $$R_{t}=\frac{TMP}{\mu J_{s}}$$

where *R_t_* is the total membrane resistance (m^−1^); TMP is the transmembrane pressure (Pa); *μ* is the viscosity of the permeate, approximated as pure water (Pa·s); and *J_s_* is the stable permeate flux (m·s^−1^).

The membrane intrinsic resistance, *R_m_*, is defined by Equation (5):

(5) $$R_{m}=\frac{TMP}{\mu J_{0}}$$

where *R_m_* is the membrane intrinsic resistance (m^−1^) and *J*_0_ is the initial permeate flux (m·s^−1^).

The irreversible fouling resistance, *R_f_*, is defined by Equation (6):

(6) $$R_{f}=\frac{TMP}{\mu J_{w}}-R_{m}$$

where *R_f_* is the irreversible fouling resistance (m^−1^).

The cake-layer resistance, *R_c_*, is defined by Equation (7):

(7) $$R_{c}=R_{t}-R_{m}-R_{f}$$

where *R_c_* is the cake-layer resistance (m^−1^).

Therefore, the total resistance can be expressed as:

(8) $$R_{t}=R_{m}+R_{c}+R_{f}$$

In this study, *J*_0_ and *J_s_* were obtained from the filtration experiments under continuous aeration, and *J_w_* was measured after cleaning.

The system recovery, R, is defined by Equation (9):

(9) $$R=\frac{Q_{p}}{Q_{f}}\times 100\%=\frac{Q_{p}}{Q_{p}+Q_{c}}\times 100\%$$

where *R* is the system recovery (%); *Q_p_* is the permeate flow rate (m^3^·h^−1^); *Q_f_* is the feed flow rate (m^3^·h^−1^); and *Q_c_* is the concentrate/sludge flow rate (m^3^·h^−1^). The water mass balance is *Q_f_* = *Q_p_* + *Q_c_*.

### 2.5. Membrane Fouling Model

Membrane fouling blocking models are tools that describe, mathematically or physically, the accumulation of foulants on the membrane surface and within membrane pores, thereby explaining the behavior of membrane flux decline. According to the relative size of foulant particles to membrane pores, they are classified into four types: complete blocking, standard blocking, intermediate blocking, and cake-layer blocking models. Details are given in Table 3, and the blocking models are shown in Figure 5.

By comparing the flux decline data obtained during actual operation with the theoretical model curves, the prevailing membrane fouling type can be identified, which helps to locate the fouling sources. Then, based on the model equations and parameters such as the initial flux and foulant concentration, the membrane flux at different times can be calculated, allowing the membrane cleaning and replacement cycles to be determined in advance.

## 3. Results and Discussion

### 3.1. Optimization of Direct Filtration Parameters

#### 3.1.1. Effect of Suspended Solids Concentration on PolyCera Membrane Direct Filtration Performance

Suspended solids (SS) concentration is one of the key water quality parameters affecting membrane filtration behavior, directly determining the formation rate of the membrane surface fouling layer and the filtration resistance. Under fixed transmembrane pressure (0.2 MPa) and aeration rate (10 L/min), the effects of influent SS concentration in the range of 300–5000 mg/L on the specific flux, produced water turbidity, and system recovery were investigated. The results are shown in Figure 6.

Figure 6a shows that under all suspended solids concentration conditions, the specific flux gradually declined with operating time, and the decline rate was positively correlated with SS concentration. In the low-concentration range (300–1000 mg/L), the curve was relatively flat, whereas in the high-concentration range (3000–5000 mg/L), the decline was accelerated in the initial stage and then gradually stabilized. This indicates that the cake layer gradually reached a dynamic equilibrium under aeration shear, and no sharp decline in specific flux occurred under any suspended solids concentration condition. Figure 6b shows that the produced water turbidity remained stably below 1.0 NTU over the entire concentration range, and the recovery decreased only slightly with increasing SS concentration. Therefore, the direct filtration equipment could still maintain stable and efficient direct filtration performance under a high suspended solids concentration shock of 5000 mg/L.

#### 3.1.2. Effect of Transmembrane Pressure on PolyCera Membrane Direct Filtration Performance

Transmembrane pressure is a key operating parameter determining the membrane filtration driving force and fouling layer behavior, directly affecting membrane flux, pollutant rejection efficiency, and system energy consumption. Under fixed influent SS concentration (5000 mg/L) and aeration rate (10 L/min), the effects of Transmembrane pressure in the range of 0.10–0.30 MPa on the specific flux, produced water turbidity, and system recovery were investigated. The results are shown in Figure 7.

Figure 7a shows that under all pressure conditions, the specific flux gradually declined with operating time, and the decline rate was positively correlated with Transmembrane pressure. When the Transmembrane pressure was 0.10, 0.15, 0.20, and 0.25 MPa, the specific flux declined gently and gradually stabilized in the later stage of operation. When the Transmembrane pressure reached 0.30 MPa, the specific flux declined sharply in the initial stage and decreased to below 0.5 in the later stage, indicating severe membrane fouling. Figure 7b shows that under all Transmembrane pressures, the produced water turbidity remained stably below 1.0 NTU and reached its minimum at 0.25 MPa. The recovery increased first and then leveled off with increasing pressure, and the increase at 0.30 MPa was smaller than that at 0.25 MPa. Considering the specific flux decline rate, the lowest produced water turbidity, and energy consumption, 0.25 MPa was determined as the selected Transmembrane pressure.

#### 3.1.3. Effect of Aeration Rate on PolyCera Membrane Direct Filtration Performance

Aeration rate is a key operating parameter for controlling membrane surface fouling in submerged membrane processes, and the gas–liquid two-phase flow shear force it generates directly affects the structure and thickness of the cake layer as well as the filtration resistance. Under fixed influent SS concentration (5000 mg/L) and Transmembrane pressure (0.25 MPa), the effects of aeration rate in the range of 0–15 L/min on the specific flux, produced water turbidity, and system recovery were investigated. The results are shown in Figure 8.

Figure 8a shows that without aeration, the specific flux declined rapidly. At an aeration rate of 5 L/min, bubble scouring effectively alleviated the flux decline. At 10 L/min, the shear force was sufficient to control particle deposition, and the specific flux remained stable above 0.6 in the later stage. At 15 L/min, the increase in specific flux compared with that at 10 L/min was limited, indicating that the scouring effect had reached saturation, while the energy consumption was higher than that at 10 L/min. Figure 8b shows that at low aeration rates, the turbidity was relatively high but still below 1.0 NTU, and it tended to stabilize after 10 L/min. The recovery increased first and then stabilized with increasing aeration rate. Considering both the specific flux control effect and aeration energy consumption, 10 L/min was determined as the selected aeration rate.

To further quantify the effect of aeration on membrane fouling, the fouling resistances at different transmembrane pressures were calculated from the stable flux obtained under continuous aeration. The results are summarized in Table 4.

As shown in Table 4, R_t_ increased with increasing transmembrane pressures, indicating that a higher driving force promoted foulant accumulation on the membrane surface. Both the cake resistance R_c_ and the irreversible resistance R_f_ increased with transmembrane pressures, suggesting that increasing transmembrane pressures aggravated both reversible and irreversible fouling. However, the contribution of R_f_ to R_t_ remained small, whereas R_c_ was the dominant fouling component. This is mainly attributed to the aeration applied during filtration. The gas–liquid two-phase flow generated by aeration produced strong shear force and turbulent disturbance near the membrane surface, which effectively removed the reversible cake layer and prevented its excessive accumulation. As a result, the irreversible fouling proportion remained low. At the selected transmembrane pressure of 0.25 MPa, the cake layer was the main reversible fouling component, while irreversible fouling contributed only a small fraction of the total resistance. When transmembrane pressures increased to 0.30 MPa, Rt increased more sharply and the stable flux declined, indicating severe membrane fouling. These results confirm that aeration is effective for controlling reversible cake-layer fouling, while periodic chemical cleaning is still necessary to remove the irreversible fouling that becomes more severe at higher transmembrane pressures.

### 3.2. Membrane Fouling Mechanism and Cleaning Strategy

#### 3.2.1. Membrane Fouling Model Fitting

To elucidate the membrane fouling mechanism, the Hermia model was used for prediction, with the coefficient of determination (R^2^) close to 1 as the fitting criterion. Under the conditions of an SS concentration of 4000–5000 mg/L, a selected pressure of 0.25 MPa, and an aeration rate of 10 L/min, the experimental data of the flat-sheet PolyCera membrane were fitted, and the results are shown in Figure 9.

The fitting results showed that the order of R^2^ values was as follows: cake-layer blocking model (0.91228) > intermediate blocking model (0.89305) > standard blocking model (0.88077) > complete blocking model (0.86659). Among the four tested models, the cake-layer blocking model gave the highest R^2^, followed by the intermediate blocking model. However, because the R^2^ values were relatively close, cake-layer blocking is proposed as the dominant mechanism rather than a fully confirmed sole mechanism. Other mechanisms, particularly intermediate blocking, may also contribute to the overall fouling behavior. This behavior is likely attributed to the synergistic effect of foulants on the membrane surface and membrane pore blocking.

#### 3.2.2. Membrane Surface Fouling Mechanism

Membrane rejection mechanism. The rejection of fine suspended solids and oil–solid composites is mainly attributed to two mechanisms. First, size exclusion by the membrane pores is the primary mechanism. The membrane pore size is much smaller than the average particle size of the suspended solids, so most fine particles are retained on the membrane surface. Second, the cake layer formed on the membrane surface acts as a secondary filtration barrier, further intercepting fine particles. The stable permeate turbidity below 1 NTU observed in this study supports the effective rejection performance of the membrane under the tested conditions.

To determine the composition of foulants on the membrane surface, complementary characterization using SEM-EDS and XRD was performed. The results are shown in Figure 10.

SEM-EDS and XRD were used to characterize the fouled membrane in order to clarify the composition of membrane surface foulants and the fouling mechanism. SEM images showed that the membrane surface was completely covered by solid particles of different particle sizes, and the particles were mixed and stacked to form a continuous and dense fouling layer. EDS detected various elements, including Si, Al, Fe, Ca, and S. These elements are consistent with the mineral composition of the raw water, such as quartz, kaolinite, calcite, and iron-containing minerals, and are therefore considered characteristic elements of the fouling layer. The XRD pattern identified several crystalline inorganic minerals, with sharp and clear diffraction peaks. Because emulsified oil is an amorphous organic substance, its signal was not directly identified by XRD. However, the SEM morphology and the observed cleaning behavior indirectly suggest the presence of organic components in the fouling layer. Based on the comprehensive characterization results, inorganic minerals formed the rigid skeleton of the fouling layer, while organic components, likely originating from coal fines and emulsified oil, filled the gaps between particles and contributed to the formation of a dense composite fouling layer.

#### 3.2.3. Membrane Cleaning Research

Based on the above fouling mechanism, the effects of different cleaning methods and cleaning agents (acidic cleaning agent: HCl; alkaline cleaning agent: NaOH; composite cleaning agent) on the flux recovery rate were investigated. Pure water backwashing increased the recovery rate from approximately 68% to 76% within 30–90 s, whereas the combined cleaning of “aeration + pure water backwashing” achieved a recovery rate of approximately 80% at 60 s and then tended to stabilize. The gas–liquid two-phase flow shear force and turbulent disturbance generated by aeration could effectively loosen the cake layer, making it easier for the backwashing water flow to detach pollutants. Accordingly, the physical cleaning method was determined to be “aeration + pure-water backwashing,” with an aeration rate of 10 L/min, a backwashing duration of 60 s, and a physical cleaning interval of 60 min.

As shown in Figure 11, the cleaning performance of single chemical agents and the composite agent was further compared. The flux recovery rates after cleaning with the acidic, alkaline, and composite agents were 84.6%, 88.4%, and 95%, respectively. SEM–EDS analysis of the membrane-surface foulants indicated that although HCl could dissolve soluble inorganic scales such as calcium carbonate [28,29], it was ineffective in removing emulsified oil. The residual oil formed an organic barrier layer on the membrane surface, hindering the penetration of the cleaning solution into deeper layers. NaOH could degrade lipid-based organic adhesive layers via saponification, but it lacked the ability to strip inert inorganic minerals such as quartz and kaolinite; consequently, substantial amounts of inorganic particles remained [30]. In contrast, the composite cleaning agent exhibited synergistic advantages. NaOH provides a strong alkaline environment, and OH^−^ reacts with esters, fatty acids, and other components in the emulsified oil through saponification and hydrolysis, producing soluble fatty acid salts and glycerol, which break down the organic binding layer. The chelating ability of HEDP contributes to the removal of inorganic scales. HEDP contains two phosphonate groups, which can dissociate to form negatively charged ligands and chelate multivalent metal ions through stable five- or six-membered chelate rings. Thereby dissolving calcium- and magnesium-containing inorganic scales and disrupting the mineral skeleton of the fouling layer, HSB is an amphoteric surfactant that contains quaternary ammonium cations and sulfonate anions. It adsorbs at the oil-water interface, reducing interfacial tension and emulsifying/dispersing oil. At the same time, it increases the hydrophilicity of the membrane surface and helps peel off oil-solid composite contamination layers. The three components synergistically penetrated into the interior of the fouling layer and simultaneously stripped organic and inorganic deposits, effectively overcoming the poor cleaning performance of a single agent. After cleaning with the composite agent, the membrane surface morphology returned to a smooth state, and the response intensities of characteristic EDS elements were greatly reduced, confirming that the composite cleaning agent was the optimal chemical cleaning agent.

#### 3.2.4. Optimization of Membrane Cleaning Using Response Surface Methodology

To improve chemical cleaning efficiency, reduce the amount of chemical agents used, and lower operating costs, response surface methodology (RSM) was employed to precisely optimize the chemical cleaning conditions. In the response surface experiments, the concentrations of the chelating agent and surfactant were kept constant, and the effects of pH, cleaning time, and cleaning temperature on membrane fouling cleaning were investigated. The selected operating conditions and ranges were as follows: pH 11.5~12.5, cleaning temperature 30~50 °C, and cleaning time 30~90 min. According to the 17 experimental schemes designed by the software, the membranes were cleaned under different cleaning conditions, and the cleaning results are shown in Table 5.

The experimental results in Table 5 were analyzed using Design-Expert 13 software, and the quadratic response regression equation relating the cleaning effect to the influencing factors was obtained as follows:

Y = 96.74 + 2.61X_1_ + 1.54X_2_ + 1.07X_3_ + 0.725X_1_X_2_ + 0.55X_1_X_3_ + 0.05X_2_X_3_ − 4.01X_1_^2^ − 2.86X_2_^2^ − 3.83X_3_^2^

As shown in Table 6, the model *p*-value was 0.0001, which is lower than 0.05, indicating that the model was statistically significant. The lack of fit was not significant (*p* = 0.0559 > 0.05). A larger F-value indicates a more significant effect of the corresponding factor. The significance order of the three factors was pH (X_1_) > temperature (X_2_) > time (X_3_). The RSM experimental data were fitted using Design-Expert software, and 3D response surface plots and corresponding contour plots were obtained (Figure 12). These plots show the relationships between the influencing factors and cleaning efficiency. The slope of the response surface reflects the degree of influence of each factor on the response (F_RR_); a steeper slope indicates a stronger effect. The contour shape is often used as an indicator of factor interactions: elliptical contours may suggest possible interactions, whereas circular contours indicate weak interactions. In this study, the contour plots exhibited elliptical characteristics, suggesting possible interactions among pH, cleaning temperature, and cleaning time. However, because the interaction terms were not statistically significant in the ANOVA (*p* > 0.05), the main effects were considered dominant. According to the response surface slopes, pH had a greater effect on F_RR_ than cleaning time or temperature, indicating that pH was the main factor affecting flux recovery. Membrane fouling is a composite layer composed of organic adhesives from emulsified oil, inorganic particles such as coal fines and rock dust, and calcium/magnesium inorganic salt precipitates. The core mechanism of alkaline chemical cleaning for removing organic oil fouling relies on the saponification hydrolysis reaction between hydroxide ions and emulsified oils. The cleaning mechanism is shown in Figure 13. The pH of the cleaning agent directly determines the effective concentration of free OH^−^ and is the decisive parameter controlling whether the saponification reaction proceeds sufficiently. Higher temperature can accelerate the diffusion of the cleaning agent by enhancing molecular thermal motion and increasing its activity. A suitable cleaning time allows the cleaning agent to fully clean the membrane surface and pores. However, excessively long cleaning time not only increases energy consumption but also causes damage to the membrane material due to prolonged exposure to an alkaline environment, thereby affecting the membrane service life.

Response surface fitting results showed that there was an obvious peak in cleaning efficiency. Through optimization calculations using Design-Expert software, the chemical cleaning conditions were determined as follows: cleaning pH 12.1, cleaning temperature 43.1 °C, and cleaning time 65 min, with a predicted cleaning efficiency of 96%. Substituting the selected conditions (pH 12.1, 43.1 °C, 65 min) into the regression equation gave a calculated cleaning efficiency of approximately 97.4%, which is close to the software-predicted value of 96%. The small difference is attributed to rounding and the quadratic approximation of the model. To verify the reliability and practicality of these predicted results, parallel validation experiments were carried out using membrane modules under the above optimal conditions. The experimental results showed that the flux recovery rates of the modules remained stably above 96%, which was in good agreement with the predicted value, further demonstrating the excellent repeatability and universality of the optimal conditions. These optimized conditions not only improved the efficiency of chemical cleaning but also reduced chemical consumption and equipment corrosion risk, while ensuring the stability of the cleaning effect.

### 3.3. Simulation and Optimization of Hydraulic Conditions of Membrane Modules

To optimize the antifouling capability of PolyCera flat-sheet membrane equipment in practical mine water treatment, the hydraulic conditions of the membrane module were investigated using Ansys Fluent 2025R2 software. For the single-phase water flow simulation (Figure 14), the standard k–ε turbulence model was used. The computational domain consisted of a hexahedral mesh with 1,137,265 cells. The boundary conditions were set as follows: pressure inlet with a gauge pressure of 0 Pa, pressure outlet with a gauge pressure of −80,000 Pa, and no-slip walls for the membrane surface and tank walls. The convergence criterion was set to residuals below 1 × 10^−4^ for continuity, momentum, turbulent kinetic energy, and turbulent dissipation rate. Convergence was further confirmed by monitoring the outlet mass flow rate and the average membrane surface velocity, which stabilized with fluctuations below 1% over the final 200 iterations. The simulation results are shown in Figure 14.

Figure 14 shows the velocity distribution contours of flat-sheet membrane direct filtration under different membrane spacings (0.5–3 cm). When the membrane spacing was 0.5–1.5 cm, the inter-membrane channel was too narrow, the fluid velocity distribution was uneven, and obvious low-velocity stagnation zones appeared in local regions, which were not conducive to the shear detachment of pollutants. As the membrane spacing increased to 2 cm, the velocity distribution within the channel gradually became uniform, and the proportion of high-velocity regions increased, indicating that an appropriate channel width was beneficial to the full development of gas–liquid two-phase flow and enhanced the scouring effect on the membrane surface. When the membrane spacing further increased to 2.5 cm and 3 cm, because the influent flow rate remained constant, the increase in flow cross-sectional area directly resulted in no increase in the overall flow velocity within the channel. The excessively low crossflow velocity weakened the scouring and detachment effect of the water flow on the membrane surface, making fine particles more prone to deposition on the membrane surface and formation of a dense cake layer, thereby increasing the filtration resistance. Although the flow channel became wider, it led to reduced scouring efficiency, and the overall equipment size increased, raising the equipment construction cost. Considering multiple factors such as scouring efficiency, anti-clogging capability, and equipment compactness, 2 cm was determined as the optimal membrane spacing.

The gas–liquid two-phase flow was modeled using the Eulerian–Eulerian model, with air as the dispersed phase and water as the continuous phase. Turbulence was modeled using the standard k–ε model. The computational domain consisted of a hexahedral mesh with 1,882,796 cells. The boundary conditions were set as follows: the aeration holes were set as velocity inlets with an air velocity of 1.7 m/s; the top of the tank was set as a pressure outlet with a gauge pressure of 0 Pa (open to atmosphere, operating pressure 101,325 Pa); the membrane surface and tank walls were set as no-slip walls. The convergence criterion was residuals below 1 × 10^−4^ for continuity, momentum, and volume fraction equations. Convergence was further confirmed by monitoring the average membrane surface shear stress and the outlet gas holdup, both of which stabilized with fluctuations below 5% over the final 200 iterations. The simulation results are shown in Figure 15.

Figure 15a compares the effect of the number of aeration pipes (2, 3, and 4) in a single membrane string on the membrane surface shear stress. The results showed that with 3 and 4 aeration pipes, the membrane surface shear stress distribution was the most uniform, and the average shear stress was higher than that with 2 aeration pipes. For the shear stress distribution with 3 aeration pipes, the shear stress distribution area with 4 aeration pipes did not increase. Considering equipment cost and size, the optimal number of aeration pipes was 3. Figure 15b compares the effect of the number of aeration holes per aeration pipe (1, 2, and 3). With 2 aeration holes, the membrane surface shear stress distribution was the most uniform and the peak region was wide. With 1 hole, bubbles were concentrated, causing excessive local scouring and insufficient scouring in other regions. With 3 holes, the shear stress distribution was not better than that with 2 holes. Considering the gas flow rate and scouring force per hole, the optimal number of aeration holes was 2. Figure 15c examines the effect of the distance between the aeration pipe and the membrane sheet (10, 20, and 30 cm). When the distance was 20 cm, the membrane surface shear stress distribution was optimal, which not only ensured that bubbles fully diffused and covered the membrane surface during ascent but also avoided concentrated impact caused by too short a distance or energy dissipation caused by too long a distance.

Based on the comprehensive CFD simulation results, the optimal design parameters of the membrane module were determined as follows: membrane spacing of 2 cm, 3 aeration pipes per membrane string, 2 aeration holes per aeration pipe, and a distance of 20 cm between the aeration pipe and the membrane sheet. This combination can achieve uniform and efficient scouring of the membrane surface at relatively low energy consumption and delay the accumulation of the cake layer.

### 3.4. Verification of Membrane Cleaning Flux Recovery

Based on the previously obtained selected operating parameters and hydraulic conditions of the membrane module, the flux recovery after membrane cleaning was verified using a submerged flat-sheet PolyCera membrane system (total effective membrane area of 100 m^2^, treatment capacity of 3 m^3^/h). The long-term validation system transmembrane pressure was intentionally reduced to 80 kPa to lower the fouling rate, extend the cleaning interval, and reduce energy consumption during long-term operation. The resulting flux of approximately 30 LMH fully satisfied the 3 m^3^/h treatment capacity.

The system was operated at a feed flow rate of 3.5 m^3^/h, corresponding to a permeate flow rate of approximately 3 m^3^/h and a concentrate flow rate of 0.5 m^3^/h, giving a recovery of approximately 85%. The results are shown in Figure 16.

The variations in membrane flux, Transmembrane pressure, and turbidity during the 86-day continuous operation are shown in Figure 16a,b. The system was operated at a treatment capacity of 3 m^3^/h. The influent suspended solids concentration ranged from 4300 to 5000 mg/L. During the 86-day operation, chemical cleaning was performed three times, on days 24, 53, and 76, respectively. The corresponding cleaning intervals were 24, 29, and 23 days. The Transmembrane pressure increased slowly and steadily with operating time, without any sharp jump, and membrane fouling was dominated by reversible cake-layer fouling. After each composite chemical cleaning, the flux recovery rate reached more than 95%. During long-term operation, the produced water turbidity remained stably below 1 NTU and was not affected by fluctuations in influent water quality.

Compared with existing mainstream processes, the direct filtration process shows the potential to replace or simplify the three-stage process of “coagulation-sedimentation-sand filtration-ultrafiltration.” Multi-stage treatment processes such as coagulation–sedimentation, sand filtration, and ultrafiltration have long flows, large footprints, and require the addition of coagulants and flocculants. In contrast, direct filtration technology can achieve efficient interception of suspended solids through the physical screening effect of precise membrane pores, shortening the multi-stage treatment process to direct filtration. This advantage was fully demonstrated in long-term verification: the direct filtration equipment consists only of a membrane module and a supporting cleaning unit, eliminating structures such as coagulation tanks, sedimentation tanks, and sand filter tanks, and reducing the footprint. In terms of operating cost, the direct filtration process avoids the addition of coagulants and flocculants such as PAC and PAM. The operating cost considered in this study includes cleaning chemicals, aeration energy, and pumping energy, but excludes membrane replacement, depreciation, and sludge disposal. Therefore, the economic assessment is limited to operating costs and does not represent a full life-cycle cost analysis. Under the tested conditions, the proposed process showed a potential operating-cost advantage over the conventional coagulation–sedimentation process; however, because the cost boundaries may differ, a site-specific cost analysis is still required before full-scale application. In addition, the system has a high degree of automation and may facilitate reduced labor and management costs. In summary, the direct filtration process shows potential to reduce chemical consumption, footprint, and operating cost while maintaining stable effluent quality, and therefore has promising technical and economic prospects.

## 4. Conclusions

The suspended solids in mine water with high-concentration fine suspended solids are dominated by fine particles (<10 μm). The zeta potential is −30 to −40 mV, and the isoelectric point is far from natural pH. Minerals such as quartz and kaolinite in rock dust provide high negative charge, and the hydrophobic surfaces of coal fines adsorb emulsified oil to form oil–solid composites. The synergistic action of these three components makes both gravity settling and spontaneous aggregation difficult to proceed effectively. The selected operating conditions of the direct filtration equipment were a transmembrane pressure of 0.25 MPa and an aeration rate of 10 L/min. Under these conditions, when the influent SS concentration was 5000 mg/L, the specific flux remained stable, and the produced water turbidity was always below 1 NTU. Membrane fouling was dominated by cake-layer blocking. Inorganic minerals formed the rigid skeleton of the fouling layer, and emulsified oil likely acted as an organic binding medium that filled the interparticle voids and coated particle surfaces, binding discrete pollutants into a dense composite fouling layer. The combined strategy of “aeration + pure water backwashing” physical cleaning and composite cleaning agent chemical cleaning could effectively restore membrane flux. An 86-day long-term operation verification showed that the Transmembrane pressure did not exhibit a sharp jump, and the produced water quality stably met standards. Chemical cleaning was performed three times on days 24, 53, and 76, and the flux recovery rate remained above 95% after each cleaning. The direct filtration process shows the potential to simplify the conventional “coagulation–sedimentation–sand filtration–ultrafiltration” train under the tested conditions, although a full “one replaces three” replacement would require further site-specific validation. It has a relatively small footprint and a high degree of automation, and may reduce treatment cost. These results provide support for the resource utilization of mine water with high-concentration fine suspended solids and suggest promising prospects for further promotion and application, although site-specific water quality and operational conditions should be considered.

## 5. Outlook

This study has several limitations due to limited sample availability. Specifically, the permeate oil and suspended solids concentrations were not quantified, and direct evidence for the role of emulsified oil as an organic binding medium in the fouling layer was not obtained. Future work will therefore focus on filling these gaps through direct measurements of permeate oil and SS, and characterization of the fouling layer using FTIR spectroscopy and confocal laser scanning microscopy (CLSM) to elucidate the distribution and binding state of oil. Clean-membrane EDS analysis will also be performed to differentiate membrane-derived signals from foulant-derived signals. Replicate lab-scale experiments will be conducted to provide statistical variability. These efforts will not only verify the proposed mechanisms but also facilitate the reliable and sustainable implementation of the direct filtration process.

## Figures and Tables

**Figure 1:**
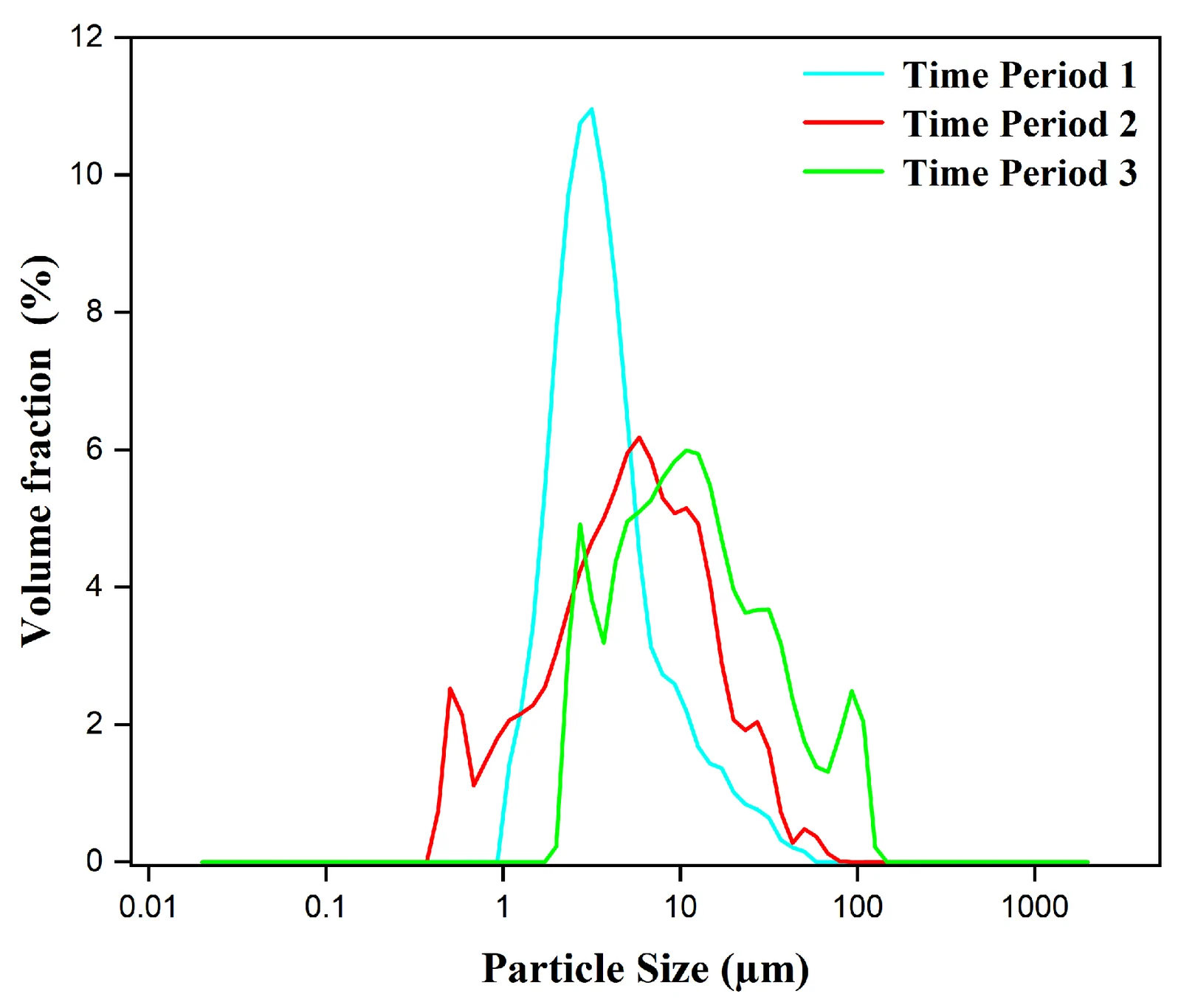
Particle size distribution during periods of different mining intensities.

**Figure 2:**
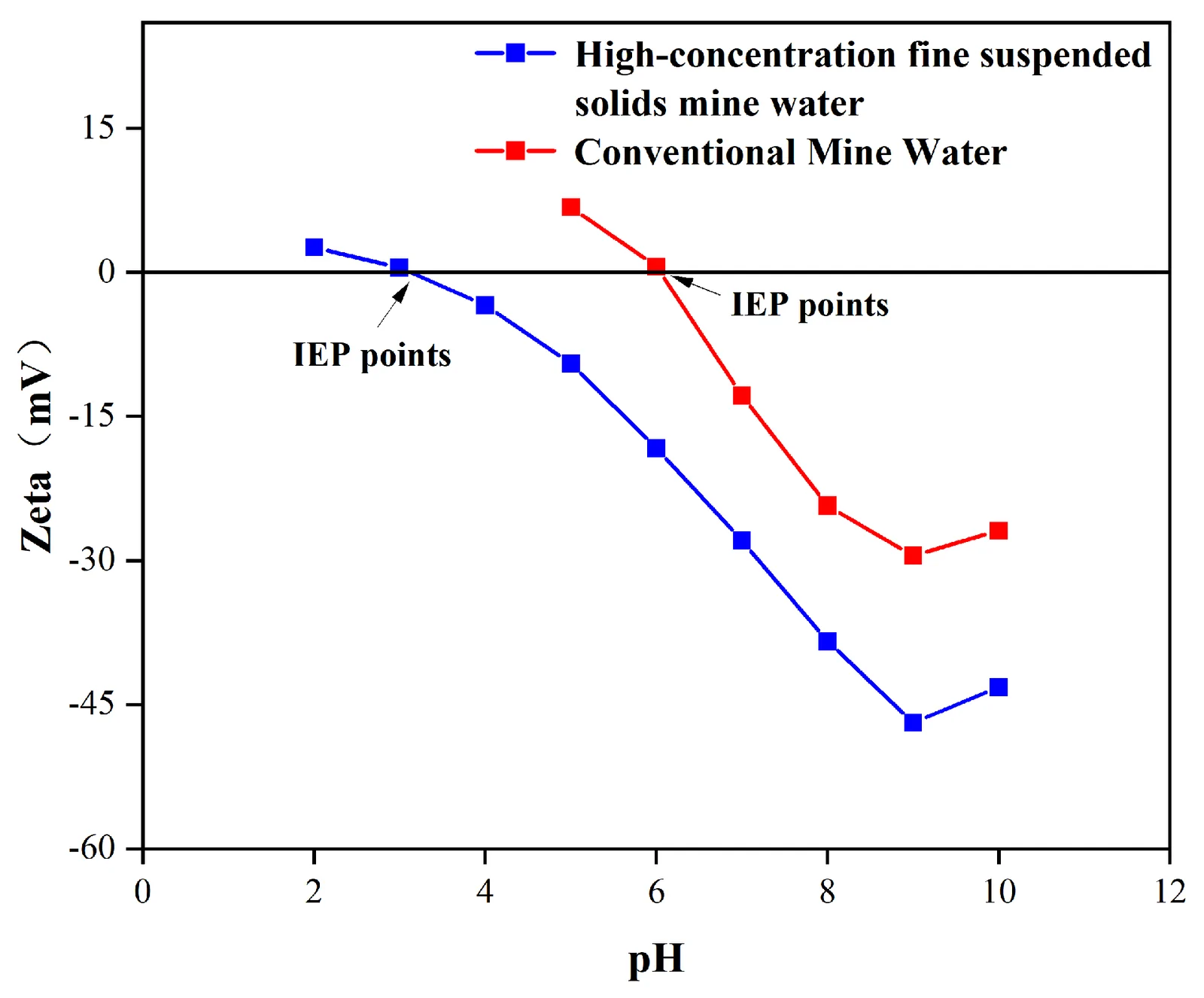
pH-zeta potential.

**Figure 3:**
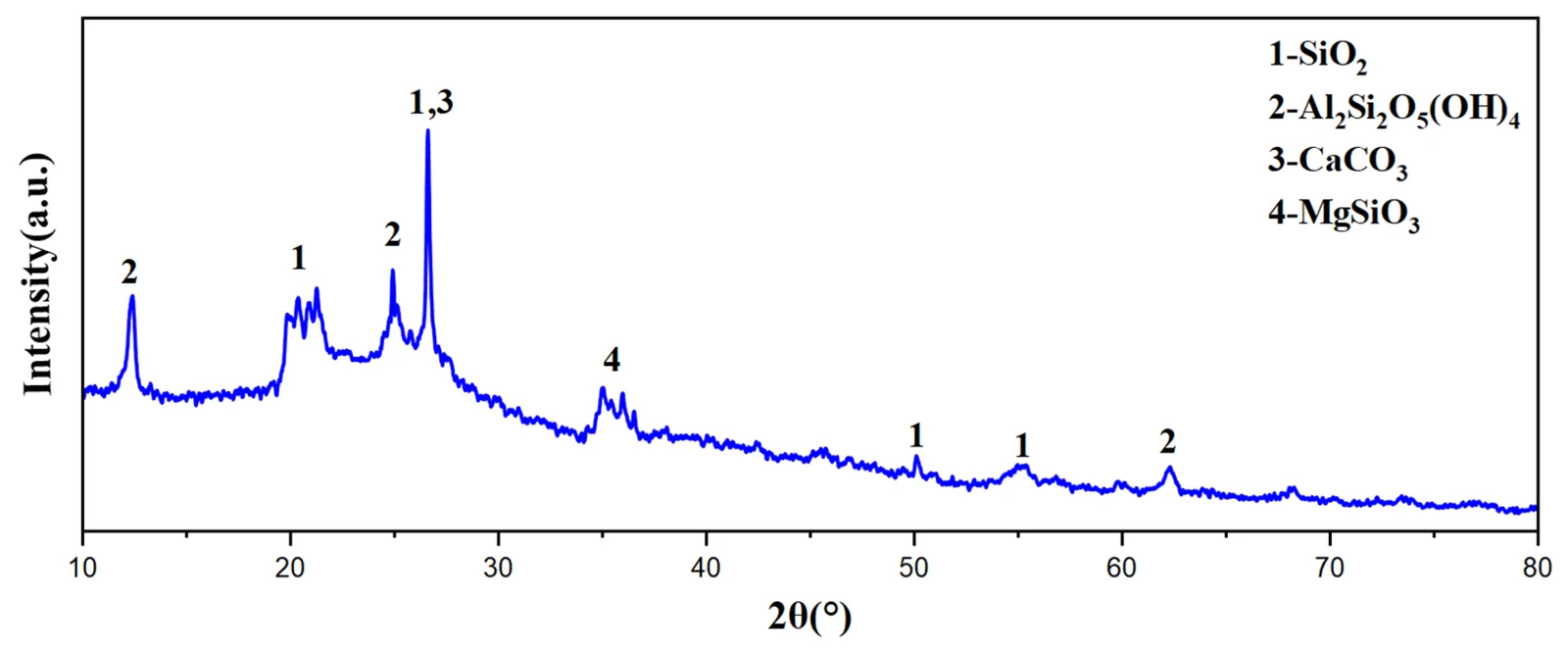
Raw water XRD analysis.

**Figure 4:**
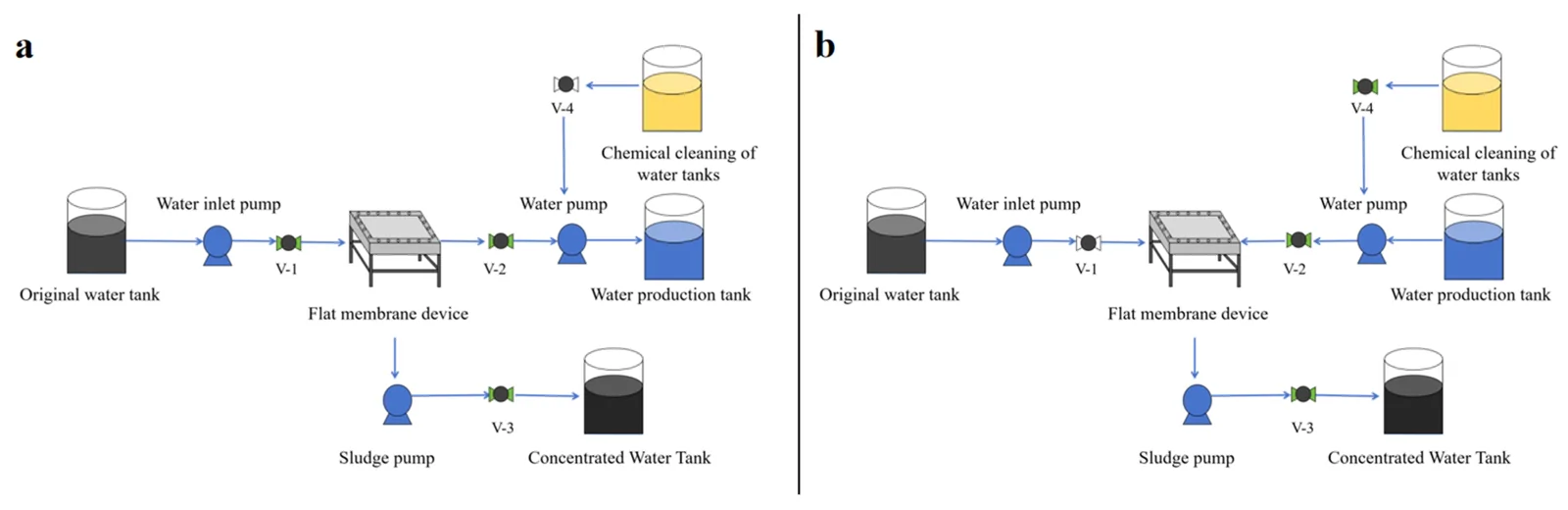
(**a**) Schematic diagram of the water production operation of the experimental device. (**b**) Schematic diagram of the cleaning operation of the experimental device. V-1: Inlet valve; V-2: Water production valve; V-3: Sludge discharge valve; V-4: Cleaning valve.

**Figure 5:**
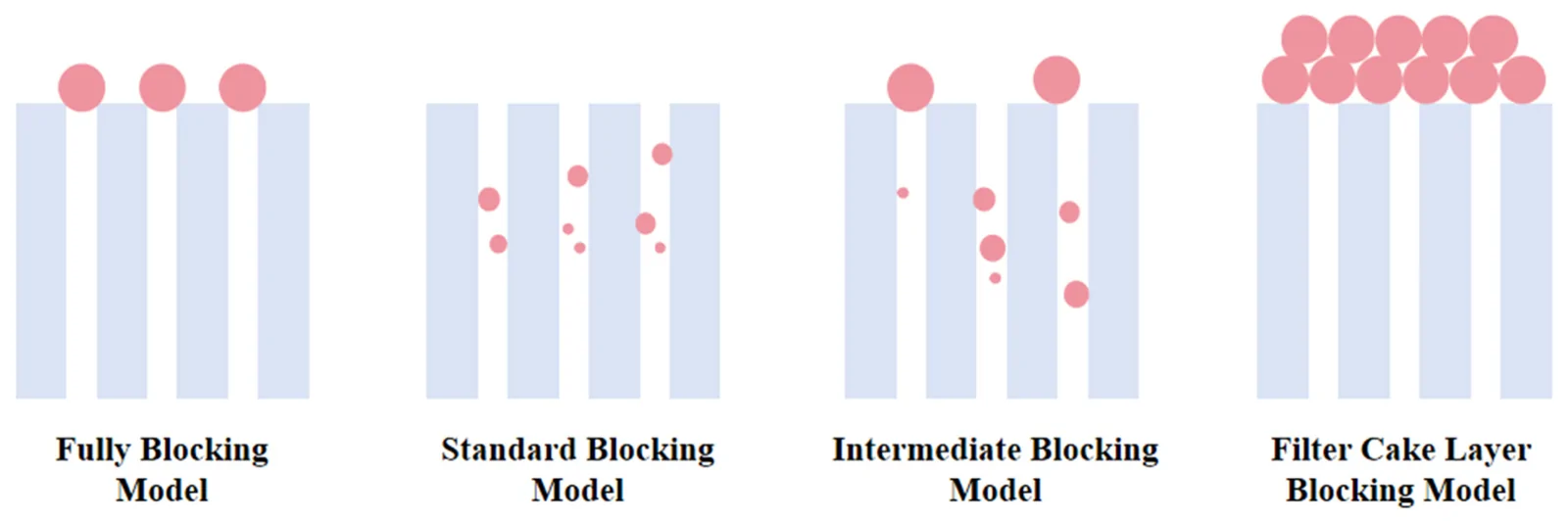
Membrane Fouling and Blocking Model.

**Figure 6:**
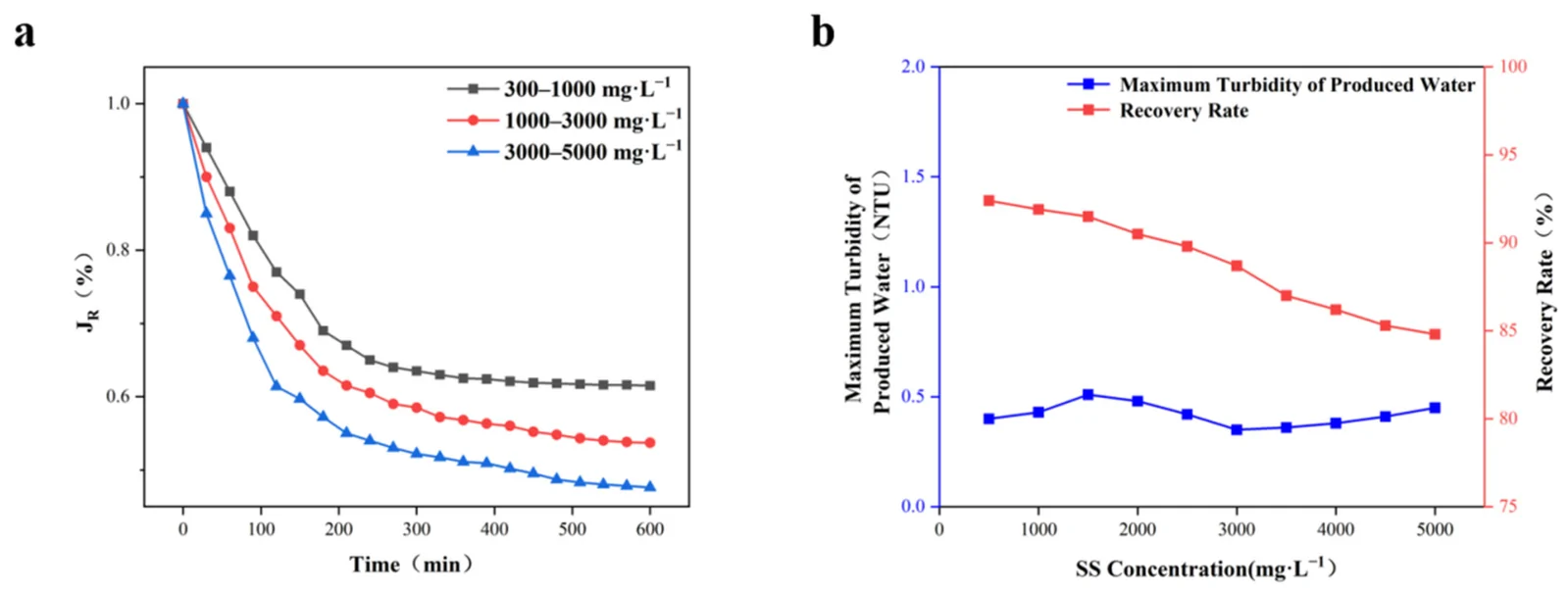
(**a**) Effect of different suspended solids concentrations on membrane specific flux. (**b**) Effect of different suspended solids concentrations on permeate turbidity and recovery rate.

**Figure 7:**
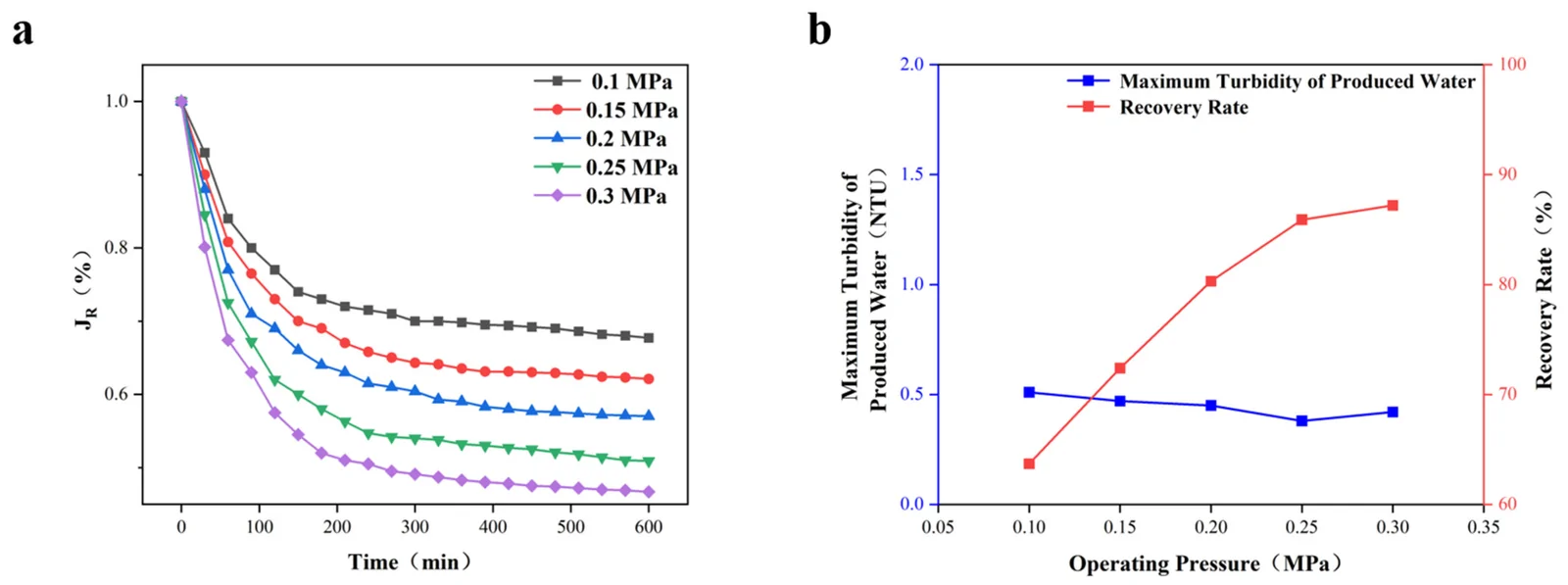
(**a**) Effect of different Transmembrane pressures on membrane flux. (**b**) Effect of different Transmembrane pressures on product water turbidity and recovery rate.

**Figure 8:**
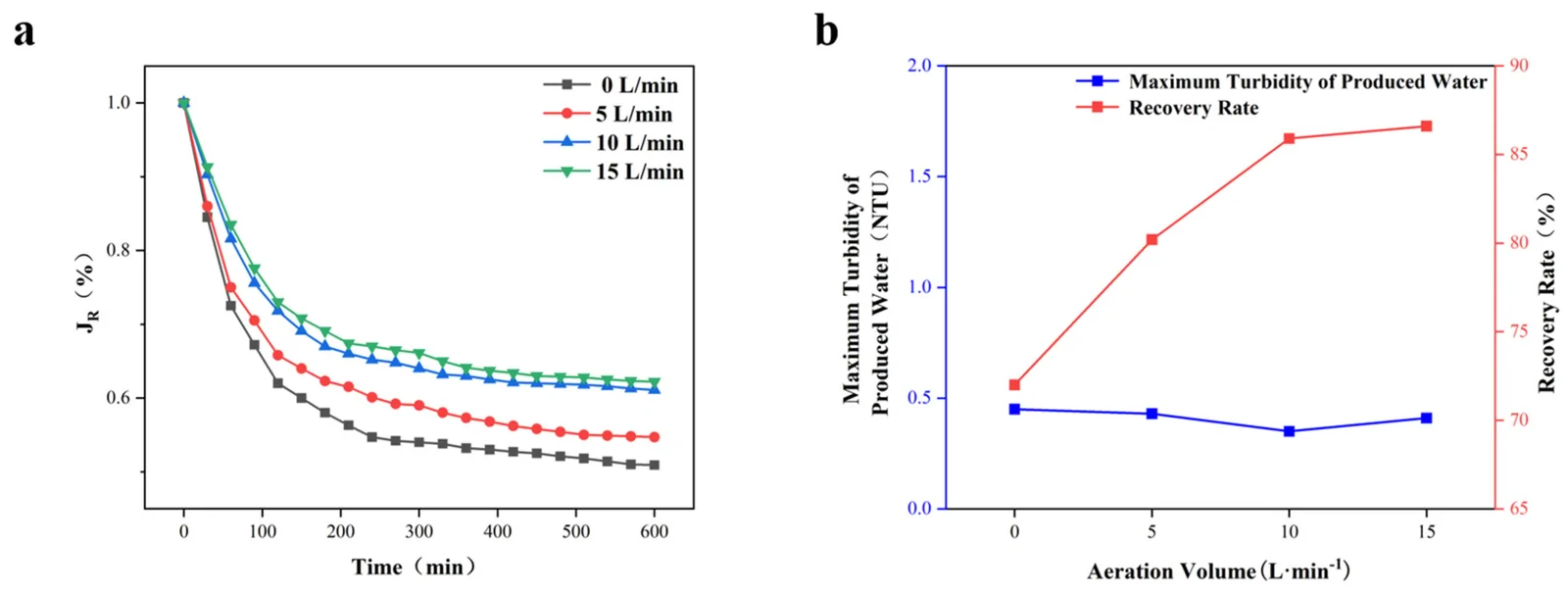
(**a**) Effect of different aeration rates on membrane flux. (**b**) Effect of different aeration rates on produced water turbidity and recovery rate.

**Figure 9:**
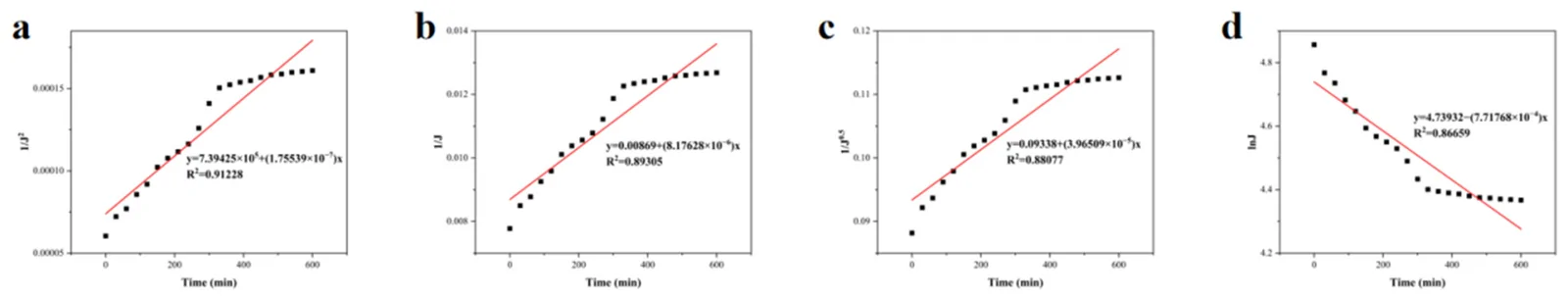
(**a**) Cake-layer blocking model. (**b**) Intermediate blocking model. (**c**) Standard blocking model. (**d**) Complete blocking model.

**Figure 10:**
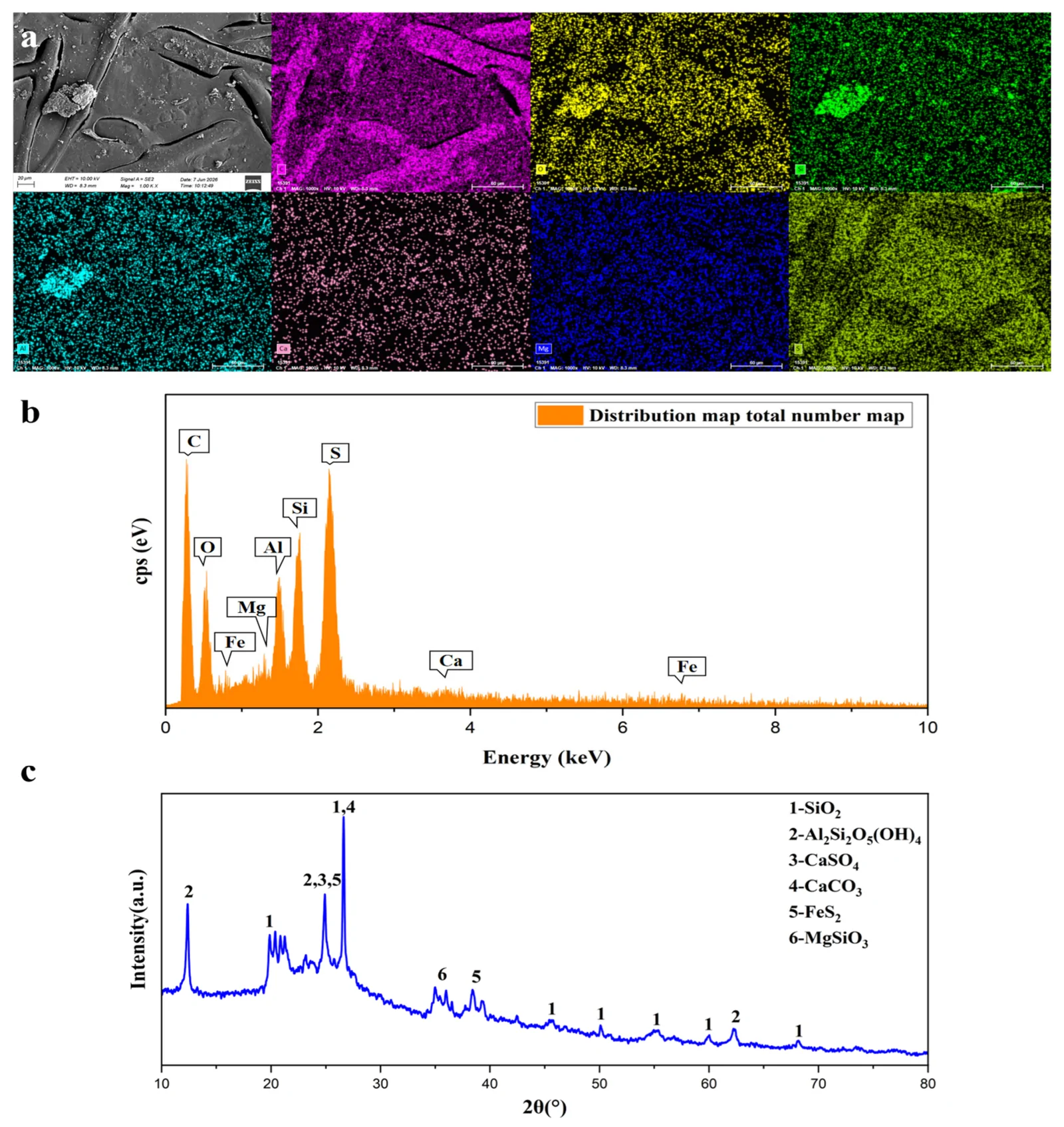
(**a**) Membrane surface SEM. (**b**) EDS characterization. (**c**) XRD characterization.

**Figure 11:**
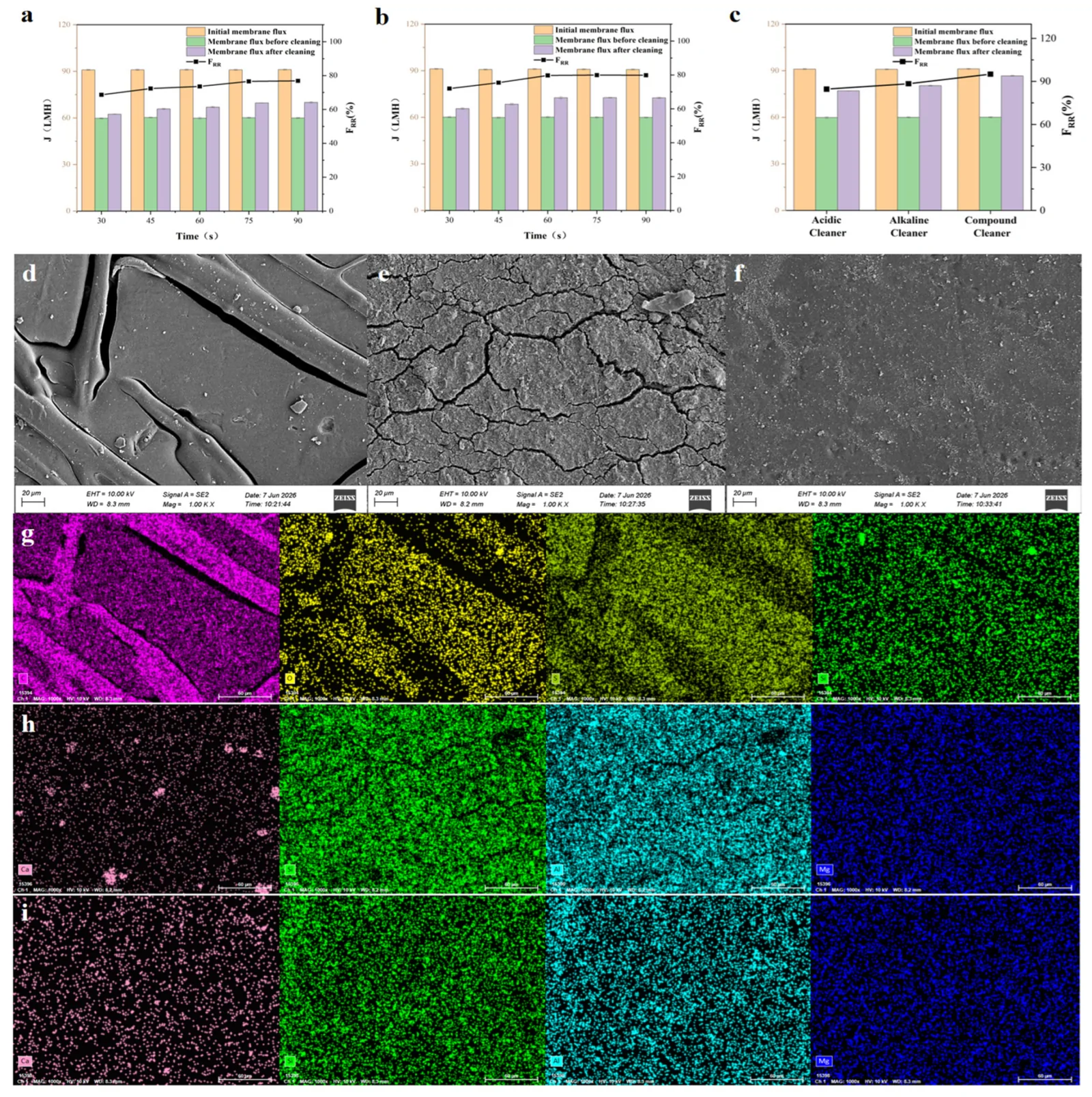
(**a**) Effect of pure water backwashing on flux recovery. (**b**) Effect of aerated pure water backwashing on flux recovery. (**c**) Effect of chemical cleaning on flux recovery. (**d**–**f**) are SEM analyses after cleaning with acidic, alkaline, and composite cleaners, respectively. (**g**–**i**) are EDS analysis results after cleaning with acidic, alkaline, and composite cleaners, respectively.

**Figure 12:**
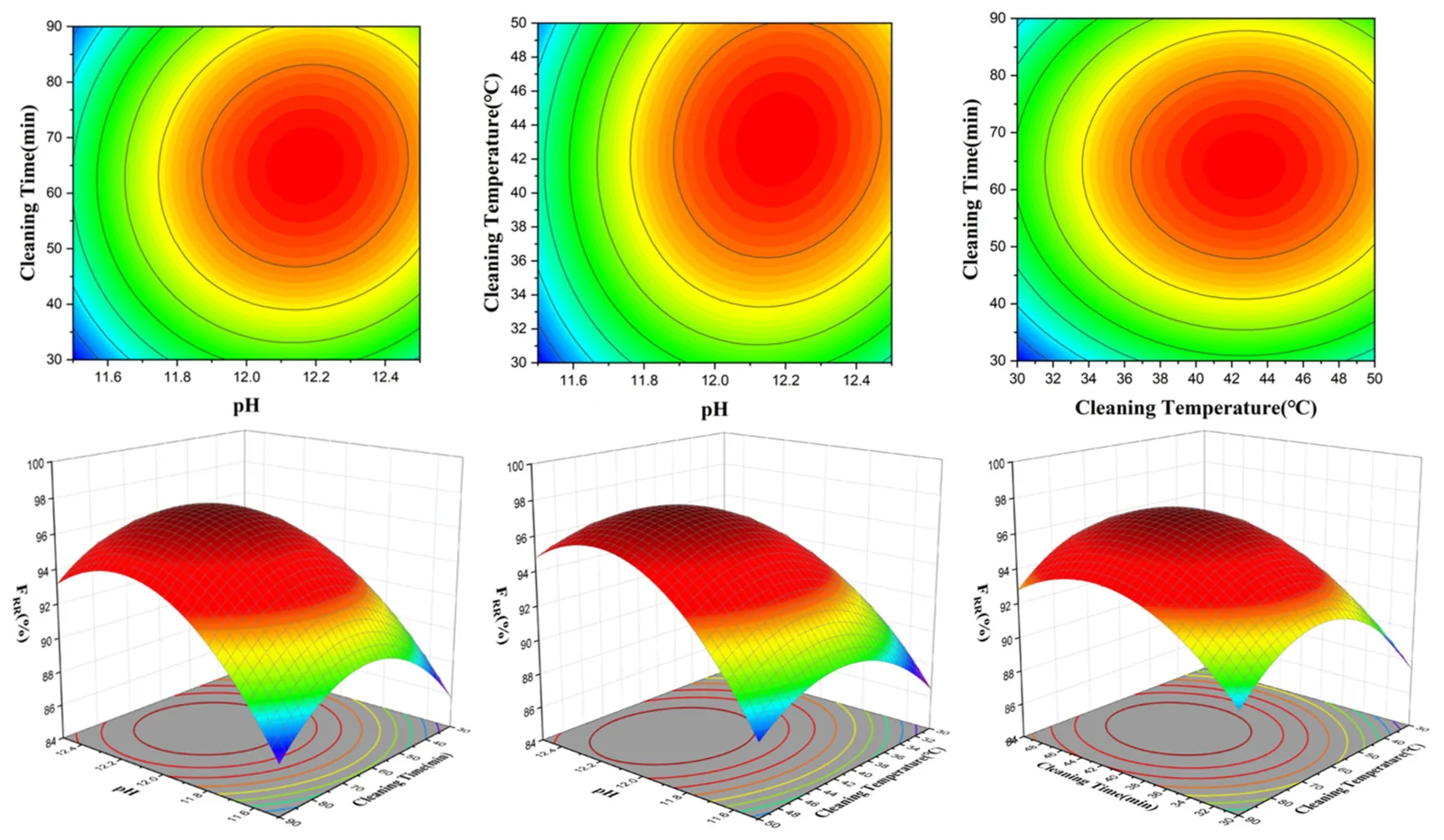
Response surface and contour plot.

**Figure 13:**
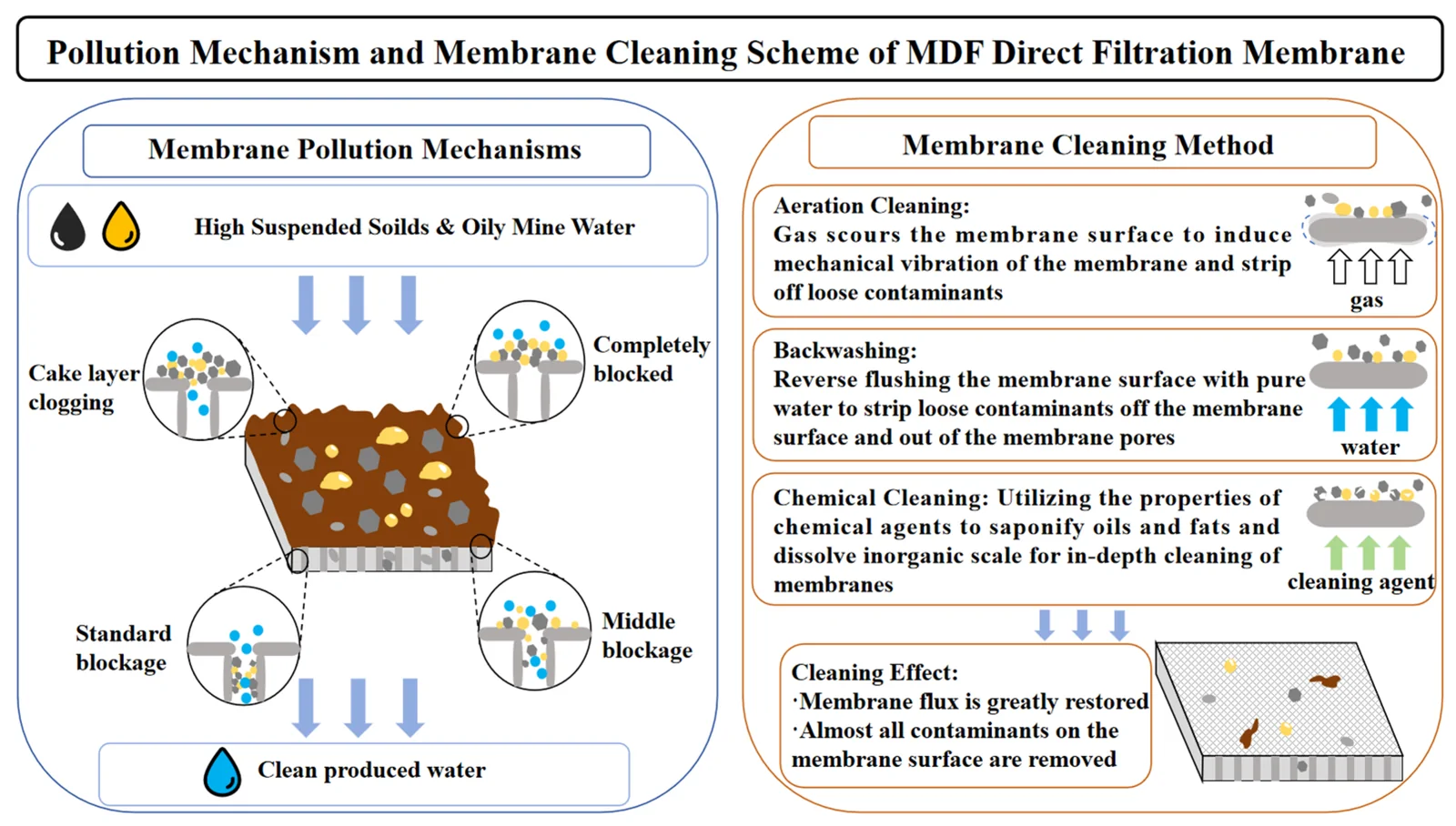
Cleaning mechanism of compound cleaning agent.

**Figure 14:**
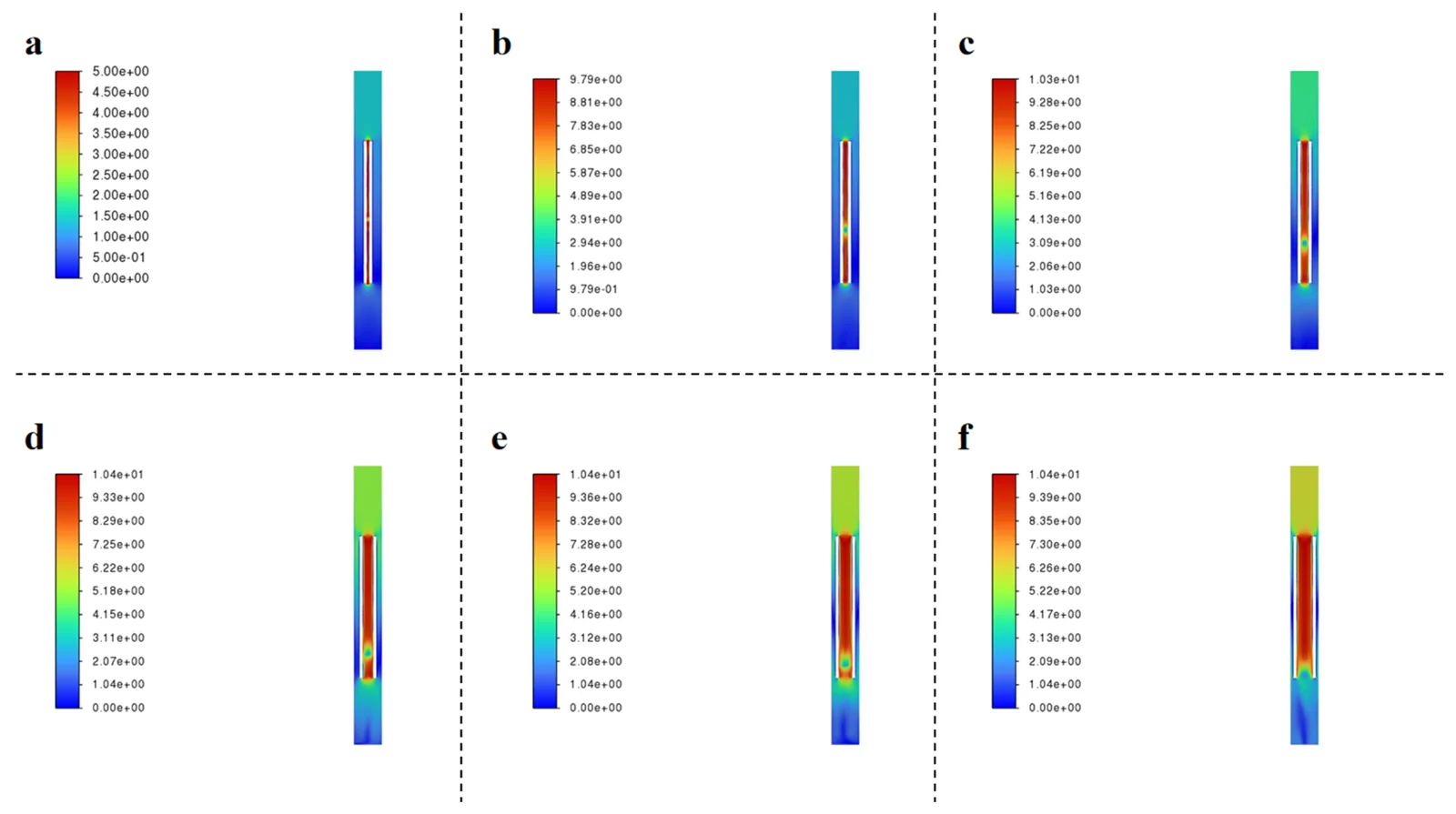
(**a**) Velocity distribution of the flat membrane at a membrane spacing of 0.5 cm. (**b**) Velocity distribution of the flat membrane at a membrane spacing of 1 cm. (**c**) Velocity distribution of the flat membrane at a membrane spacing of 1.5 cm. (**d**) Velocity distribution of the flat membrane at a membrane spacing of 2 cm. (**e**) Velocity distribution of the flat membrane at a membrane spacing of 2.5 cm. (**f**) Velocity distribution of the flat membrane at a membrane spacing of 3 cm.

**Figure 15:**
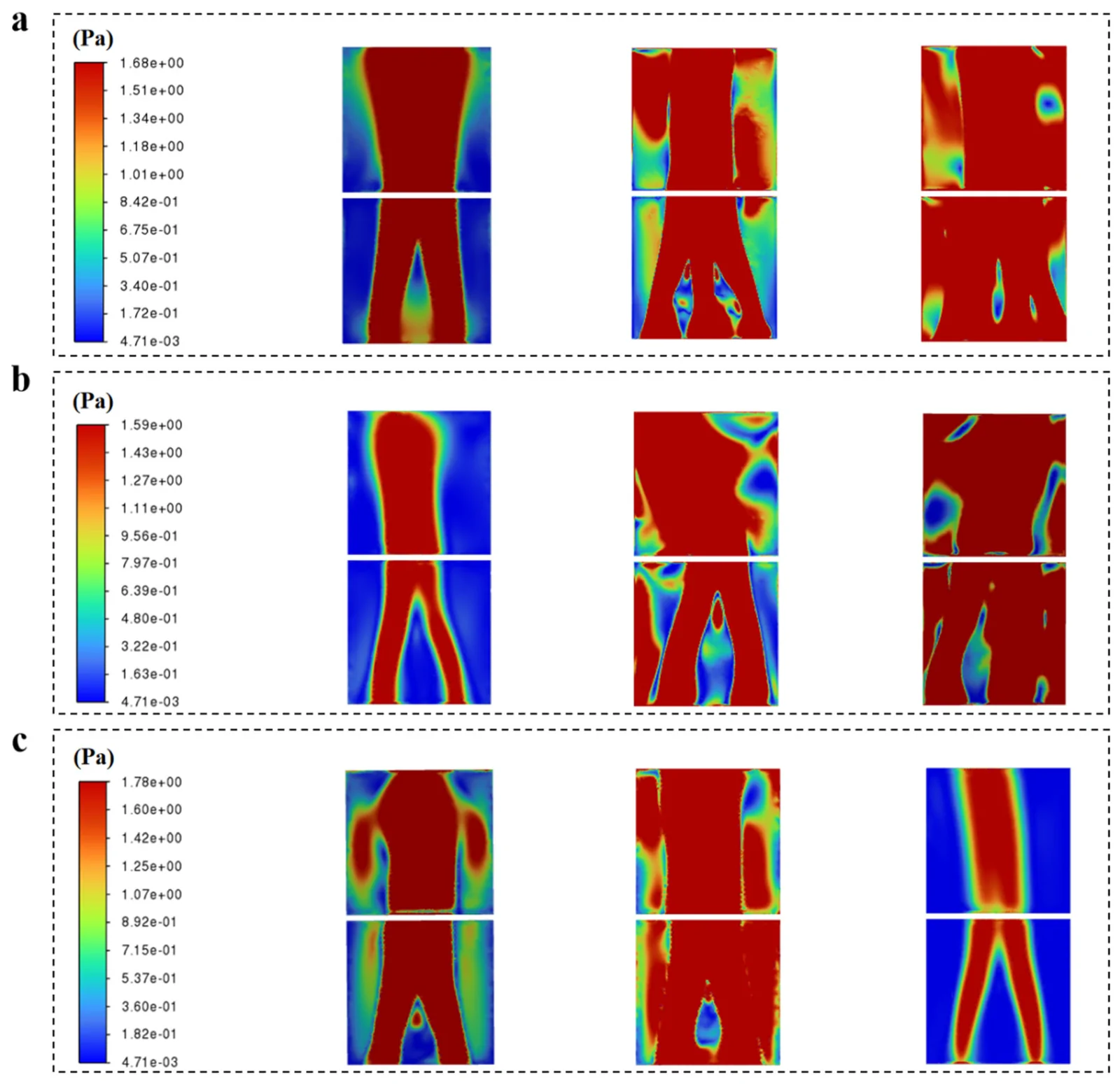
(**a**) Distribution of membrane surface shear stress when the number of downflow aeration pipes in a single membrane string is 2, 3, and 4. (**b**) Distribution of membrane surface shear stress when the number of aeration holes per pipe is 1, 2, and 3. (**c**) Distribution of membrane surface shear stress when the distance between the aeration pipe and membrane sheet is 10, 20, and 30 cm.

**Figure 16:**
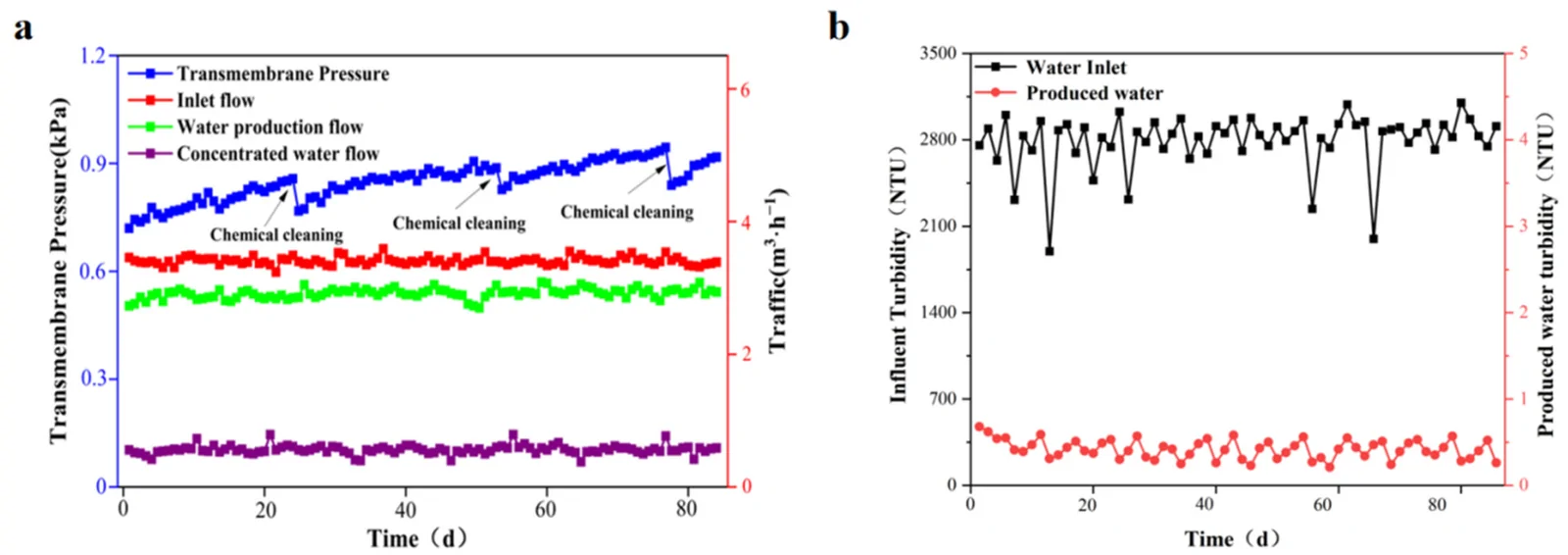
(**a**) Operational stability during 86 days of continuous operation. Chemical cleaning was performed on days 24, 53, and 76. (**b**) Direct filtration effect. The influent turbidity fluctuated, while the produced water turbidity remained below 1 NTU.

**Table 1:** Test water quality.

Water Sample	pH	SS (mg·L^−1^)	Zeta Potential (mV)	Oil (mg·L^−1^)	Hardness (mg·L^−1^)	Average Particle Size (μm)
Raw water	7.81~8.07	300~5000	−30~−40	3~5	100~600	5~10

**Table 2:** Proportion of each component in suspended matter.

Component	Unit	Proportion
Coal fines	(%)	44.3
Rock dust	(%)	36.1
Fibers	(%)	15.7

**Table 3:** Characteristics of Membrane Fouling and Blocking Models and Their Applicable Scenarios.

Model	Key Characteristics	Applicable Conditions	*n* Value	Model Equation
Complete blocking model	Foulant particle size ≥ membrane pore size; foulants directly block the pore entrances, and the blocked pores completely lose water permeability.	Treatment of feeds containing large particles, such as coarse filtration for removing suspended impurities.	*n* = 2	$lnJ=lnJ_{0}-k_{1}t$
Standard blocking model	Foulant particle size < membrane pore size; foulants enter the pores and deposit uniformly on the pore walls, gradually reducing the pore cross-sectional area.	Treatment of feeds containing small molecules and colloids, such as ultrafiltration for removing proteins and nanoparticles.	*n* = 1.5	$\frac{1}{\sqrt{J}}=\frac{1}{\sqrt{J_{0}}}+k_{2}t$
Intermediate blocking model	Foulant particle size slightly smaller than membrane pore size; some foulants block the pore entrances and some enter the pores, representing a transition state between complete blocking and standard blocking.	Feeds with a wide foulant particle size distribution, containing both particles close to the membrane pore size and smaller particles.	*n* = 1	$\frac{1}{J}=\frac{1}{J_{0}}+k_{3}t$
Cake-layer blocking model	Foulant particle size much smaller than membrane pore size; a large amount of foulants accumulates on the membrane surface to form a “cake layer,” which becomes a new filtration barrier.	Treatment of feeds with high concentrations of fine particles, such as microfiltration of high-turbidity water, where a sludge cake forms on the membrane surface.	*n* = 0	$\frac{1}{J^{2}}=\frac{1}{{J_{0}}^{2}}-k_{4}t$

where J is the membrane flux at time t (L·(m^2^·h)^−1^); J_0_ is the initial membrane flux (L·(m^2^·h)^−1^); t is the operating time (h); k_1_ is the complete blocking rate constant (m^2^·h·L^−1^), which is positively correlated with foulant concentration; k_2_ is the standard blocking rate constant (1·h^−1^), which is related to membrane pore size and foulant particle size; k_3_ is the intermediate blocking rate constant ((m·h)^1/2^·L^−1^), which is related to foulant particle size distribution; and k_4_ is the cake-layer formation rate constant (L·(m^2^·h^2^)^−1^), which is related to feed concentration and particle settleability.

**Table 4:** Transmembrane pressures, permeate flux, and fouling resistance at different operating pressures.

Transmembrane Pressures (MPa)	J_0_ (LMH)	Stable J (LMH)	R_m_ (×10^12^ m^−1^)	R_t_ (×10^13^ m^−1^)	R_f_ (×10^11^ m^−1^)	R_c_ (×10^12^ m^−1^)
0.1	55.2	38.6	6.52	0.933	3.43	2.46
0.15	71.1	45.2	7.59	1.20	4.00	3.95
0.2	88.8	55.7	8.11	1.29	4.27	4.39
0.25	110.3	65.1	8.16	1.38	4.30	5.23
0.3	128.6	72.2	8.40	1.50	4.42	6.12

**Table 5:** BBD Experimental Design and Results.

Number	X1	X2	X3	F_RR_
1	12	30	90	89.7
2	12	50	30	90.3
3	12	40	60	96.3
4	12	40	60	97.2
5	12	40	60	96.4
6	12.5	40	30	89.4
7	12	40	60	96.9
8	12	30	30	87.5
9	12.5	40	90	92.5
10	11.5	30	60	85.8
11	11.5	40	90	87.3
12	12	40	60	96.9
13	11.5	40	30	86.4
14	12.5	50	60	95.4
15	12	50	90	92.7
16	11.5	50	60	87.6
17	12.5	30	60	90.7

**Table 6:** Variance Analysis.

	Sum of Squares	df	Mean Square	F-Value	*p*-Value	Significance
Model	268.66	9	29.85	65.11	<0.0001	Significance
X_1_	54.60	1	54.60	119.09	<0.0001	
X_2_	18.91	1	18.91	41.25	0.0004	
X_3_	9.25	1	9.25	20.16	0.0028	
X_1_X_2_	2.1	1	2.1	4.59	0.0695	
X_1_X_3_	1.21	1	1.21	2.64	0.1483	
X_2_X_3_	0.0100	1	0.0100	0.0218	0.8868	
X_12_	67.62	1	67.62	147.48	<0.0001	
X_22_	34.38	1	34.38	74.98	<0.0001	
X_32_	61.84	1	61.84	134.88	<0.0001	
Residual	3.21	7	0.4585			
Lack of fit	2.64	3	0.8792	6.15	0.0559	Not Significance
Pure error	0.5720	4	0.1430			
Corrected total	271.87	16				

## Data Availability

The original contributions presented in the study are included in the article. Further inquiries can be directed to the corresponding author.
